# Effect of the Uganda Newborn Study on care-seeking and care practices: a cluster-randomised controlled trial

**DOI:** 10.3402/gha.v8.24584

**Published:** 2015-03-31

**Authors:** Peter Waiswa, George Pariyo, Karin Kallander, Joseph Akuze, Gertrude Namazzi, Elizabeth Ekirapa-Kiracho, Kate Kerber, Hanifah Sengendo, Patrick Aliganyira, Joy E. Lawn, Stefan Peterson, on behalf of the Uganda Newborn Study Team

**Affiliations:** 1 Department of Health Policy, Planning and Management, School of Public Health, College of Health Science, Makerere University, Kampala, Uganda; 2 Global Health, Department of Public Health Sciences, Karolinska Institutet, Stockholm, Sweden; 3 Iganga-Mayuge Health and Demographic Surveillance Site, Iganga-Mayuge, Uganda; 4 Malaria Consortium, London, UK; 5 Save the Children, Cape Town, South Africa; 6 MARCH Unit, London School of Hygiene and Tropical Medicine, London, UK; 7 International Maternal and Child Health Unit, Department of Women's and Children's Health, Uppsala University, Uppsala, Sweden

**Keywords:** community health workers, health system strengthening, kangaroo mother care, maternal care, newborn care, neonatal mortality, randomised controlled trial, Uganda

## Abstract

**Background:**

Care for women and babies before, during, and after the time of birth is a sensitive measure of the functionality of any health system. Engaging communities in preventing newborn deaths is a promising strategy to achieve further progress in child survival in sub-Saharan Africa.

**Objective:**

To assess the effect of a home visit strategy combined with health facility strengthening on uptake of newborn care-seeking, practices and services, and to link the results to national policy and scale-up in Uganda.

**Design:**

The Uganda Newborn Study (UNEST) was a two-arm cluster-randomised controlled trial in rural eastern Uganda. In intervention villages volunteer community health workers (CHWs) were trained to identify pregnant women and make five home visits (two during pregnancy and three in the first week after birth) to offer preventive and promotive care and counselling, with extra visits for sick and small newborns to assess and refer. Health facility strengthening was done in all facilities to improve quality of care. Primary outcomes were coverage of key essential newborn care behaviours (breastfeeding, thermal care, and cord care). Analyses were by intention to treat. This study is registered as a clinical trial, number ISRCTN50321130.

**Results:**

The intervention significantly improved essential newborn care practices, although many interventions saw major increases in both arms over the study period. Immediate breastfeeding after birth and exclusive breastfeeding were significantly higher in the intervention arm compared to the control arm (72.6% vs. 66.0%; *p*=0.016 and 81.8% vs. 75.9%, *p*=0.042, respectively). Skin-to-skin care immediately after birth and cord cutting with a clean instrument were marginally higher in the intervention arm versus the control arm (80.7% vs. 72.2%; *p*=0.071 and 88.1% vs. 84.4%; *p*=0.023, respectively). Half (49.6%) of the mothers in the intervention arm waited more than 24 hours to bathe the baby, compared to 35.5% in the control arm (*p*<0.001). Dry umbilical cord care was also significantly higher in intervention areas (63.9% vs. 53.1%, *p*<0.001). There was no difference in care-seeking for newborn illness, which was high (around 95%) in both arms. Skilled attendance at delivery increased in both the intervention (by 21%) and control arms (by 19%) between baseline and endline, but there was no significant difference in coverage across arms at endline (79.6% vs. 78.9%; *p*=0.717). Home visits were pro-poor, with more women in the poorest quintile visited by a CHW compared to families in the least poor quintile, and more women who delivered at home visited by a CHW after birth (73.6%) compared to those who delivered in a hospital or health facility (59.7%) (*p*<0.001). CHWs visited 62.8% of women and newborns in the first week after birth, with 40.2% receiving a visit on the critical first day of life.

**Conclusion:**

Consistent with results from other community newborn care studies, volunteer CHWs can be effective in changing long-standing practices around newborn care. The home visit strategy may provide greater benefit to poorer families. However, CHW strategies require strong linkages with and concurrent improvement of quality through health system strengthening, especially in settings with high and increasing demand for facility-based services.

Newborn survival has recently emerged as a global and national public health issue, driven in part by the fourth Millennium Development Goal (MDG) for child survival. Under-five mortalities are estimated to have reduced by 47% globally between 1990 and 2012, while deaths in the first month of life (the neonatal period) declined by 37% (1). Each year in Uganda there are an estimated 35,000 newborn deaths (1), with an additional 40,000 babies stillborn (2). The proportion of under-five deaths that occur in the neonatal period has increased, from 22% in 1990 to 33% in 2012 (1). With a growing proportion of under-five deaths occurring amongst newborns, child survival will increasingly be determined by national and global success in reducing newborn deaths.

The previous decade saw major advances in the evidence base for newborn survival, particularly following publication of *The Lancet* neonatal series in 2005 (3). Increasing frequency and reliability of estimates for neonatal mortality and cause of death have led to improved planning and efforts to track progress (3). In addition, there is a growing body of evidence for effective interventions and packages to address the three main causes of newborn mortality (intrapartum-related deaths, complications of prematurity, and severe infections) in weak health systems.

Studies from South Asia have demonstrated that interventions can be delivered cost-effectively using community health workers (CHWs), with a significant impact on neonatal mortality and newborn care practices (4–8). The early positive results of these trials culminated in development of a World Health Organization (WHO)–United Nations Children's Fund joint statement on home visits for newborn care (9). The recommendation was for three visits in the first week of life, with the first visit as soon as possible following delivery. The content of each visit should include examination of the mother and baby for danger signs and promotion of healthy behaviours, including early and exclusive breastfeeding, keeping the baby warm, hand washing, umbilical cord and skin care, identifying conditions requiring additional care, and counselling on when to take a newborn to a health facility (9).

In comparison to the context in much of sub-Saharan Africa, many of the South Asian trials took place in settings with very high baseline neonatal mortality, lower facility delivery rates, lower levels of care-seeking, and established CHW systems. Given these differences, there was a need to test whether these models of care could achieve the same outcomes locally. New studies from Ghana and South Africa have since provided evidence on adapted community-based newborn care packages in Africa, with promising results but less dramatic impact (10, 11).

In Uganda, the community component of the health system is led by volunteers under the national Village Health Team (VHT) strategy (12). The VHT strategy aims to have a team of 5–6 CHWs per village responsible for community mobilisation and preventive care. In 2008, the Ministry of Health began the process of revitalising and expanding the VHT programme to include newborn care and case management for older children, with 1–2 VHT members specifically dedicated to maternal, newborn, and child health. To provide policy makers with information on a scalable package for newborn care in this context, the Uganda Newborn Study (UNEST) was designed as a cluster-randomised controlled trial to evaluate a home visit package with health facility strengthening within existing health system constraints, with the objective of assessing the effect of the UNEST intervention on essential newborn care practices and care-seeking. This is the first article in a supplement reporting on the UNEST results.

## Methods

### Setting

UNEST was implemented in Iganga and Mayuge districts in eastern Uganda, within the Iganga-Mayuge Health and Demographic Surveillance Site (HDSS). The HDSS was established in 2004 in collaboration between the two districts, Makerere University, Uganda, and Karolinska Institutet, Sweden. The HDSS is predominately rural, comprising 65 villages and a total population of approximately 70,000 at the time of the study. Thirteen peri-urban villages form the Iganga Town Council. The main economic activity is subsistence farming. Other occupations include small-scale businesses, such as grain milling, market vending and motorcycle transport, and civil service employment. The predominant ethnic group in the HDSS is the Basoga, a Bantu-speaking group, which makes up 10% of Uganda's population. The HDSS is served by one 100-bed hospital and at least 19 government-run and private-sector health centres that offer delivery services (13). A rising proportion of women in the Central East region – over two-thirds of them – deliver at health facilities (14).

The cluster unit for the study was the village. Each of the 63 villages in the HDSS was randomly allocated to the intervention or control arm, without any stratification or matching due to the relatively large number of study units. Computer-generated restricted randomisation was done in a one-to-one ratio by an independent epidemiologist from the London School of Hygiene and Tropical Medicine. A total of 31 villages were allocated to the intervention arm and 32 to the control arm. More information on the study setting and design are available elsewhere (15).

### Participants and design

The trial included all consenting pregnant women and their newborns residing in the HDSS between September 2009 and August 2011. A team of data collectors linked to the HDSS conducted a baseline household survey to establish coverage of maternal and newborn care behaviours and practices. The baseline survey was conducted between March and August 2008 and included women with a live birth within 4 months of the survey. Information on household asset ownership, care received during pregnancy, childbirth and the postnatal period, and nutritional indicators were collected.

**Fig. 1:**
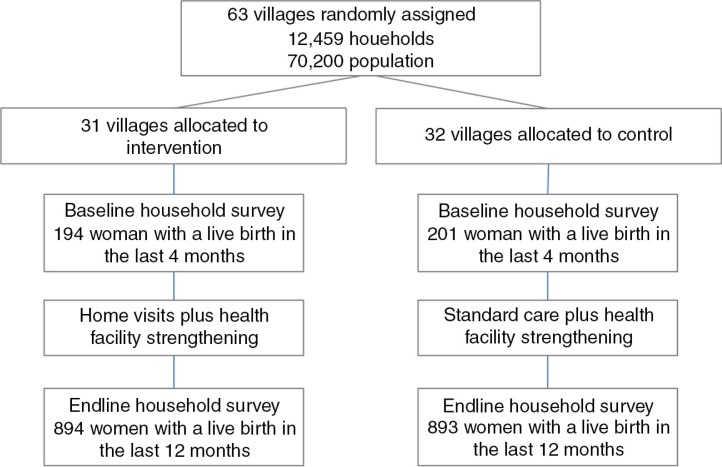
UNEST trial profile.

Data for the endline survey were collected between September and November 2011 amongst women who had a live birth within 12 months of the survey (Fig. 1). The primary outcomes of the study were improved coverage of services for antenatal care (ANC), birth preparedness, skilled attendance at delivery, and postnatal care, as well as increases in healthy practices including breastfeeding, thermal care, and hygiene. The study was not powered to detect mortality differences; however, routine birth and death reports were collected as part of household surveillance in the HDSS, but are not reported on here. Prospective data on pregnancies and their outcomes were collected between 2006 and 2010 through routine surveillance in the HDSS. In 2011, a cross-sectional pregnancy history study was conducted amongst 10,540 women aged 15–49, and details are reported elsewhere (16). Village-based scouts notified verbal autopsy interviewers of deaths, including maternal deaths, stillbirths, and neonatal deaths, as they occurred.

**Fig. 2:**
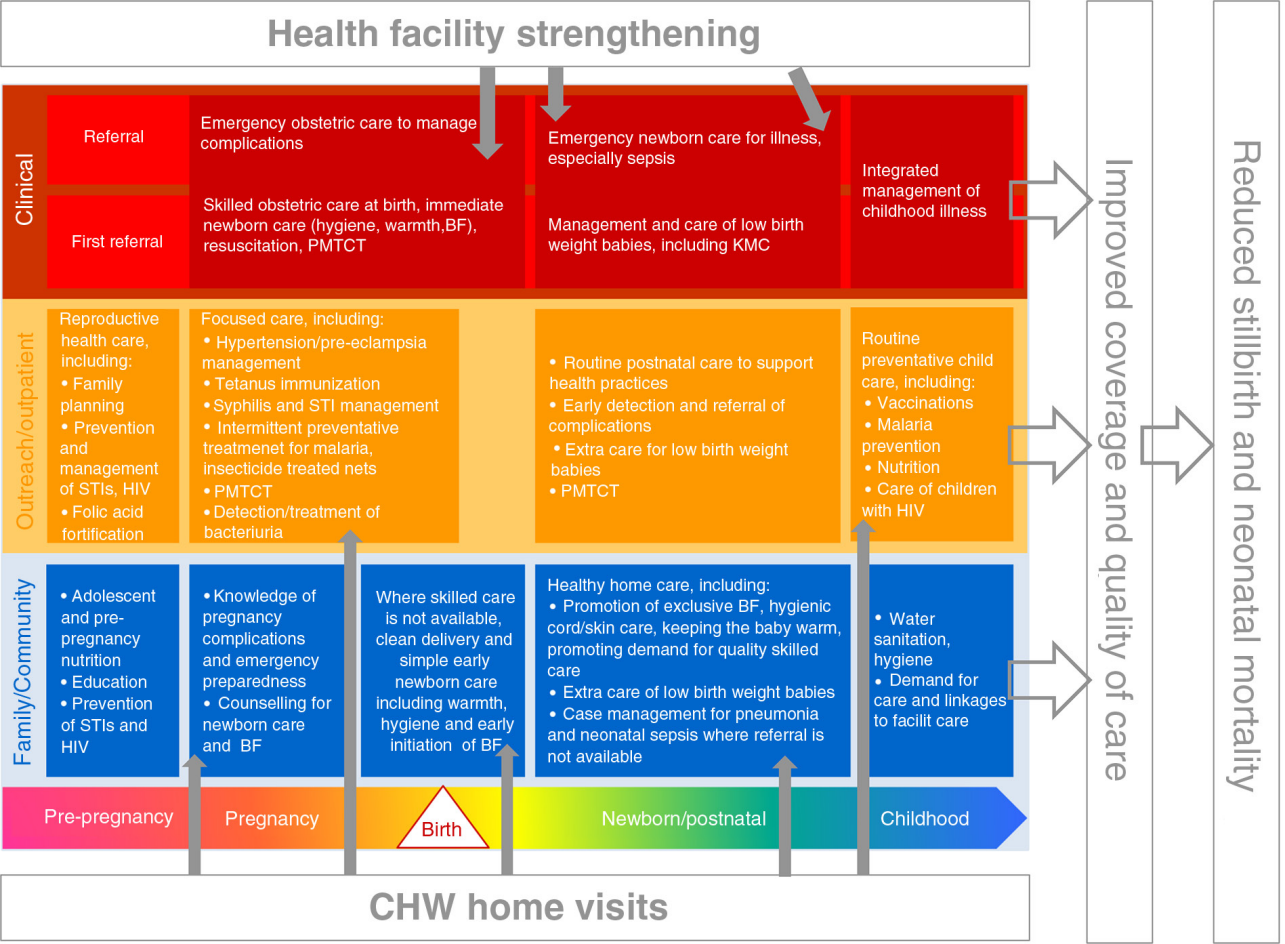
UNEST conceptual framework. Source: Adapted from Kerber et al. (22).

Meetings were held in each district in August 2008 to introduce UNEST and explain the proposed randomisation process to village members and prospective CHWs. The UNEST package was intended to be an integrated intervention package (Fig. 2), based on extensive formative research (17–22), and developed and implemented in close collaboration with national policy makers and experts and the district health management teams of the trial districts. Following a design workshop, the intervention was piloted between November 2008 and February 2009. Building on the pilot, 61 CHWs from the intervention clusters were recruited by the community with the aim of identifying individuals with the following attributes: empathy; experience of similar problems and situations; respected in the local community; and considered to be a natural helper or someone that community members would naturally go to in the event of a problem. Women were preferred, although males were also accepted (women and men can serve as VHT members according to the national strategy).

The CHWs were then trained for 5 days on the intervention package, which included identification of pregnant women in their community and undertaking two home visits during pregnancy and three visits after birth at or as close to days 1, 3, and 7 as possible. Each cluster had at least one CHW, with most villages having two, in line with the national VHT strategy (23), amounting to one CHW per 100–150 households on average. More details, including the selection, training, and supervision of CHWs as well as the content of each visit can be found in the trial protocol (15). After the initial training no additional off-site trainings were conducted, but knowledge and skills were reinforced during quarterly supervisory meetings and through directly observed supervision.

We found this strategy to be effective in imparting and retaining skills (24, 25). CHWs’ incentives were simple, and included a t-shirt, briefcase, certificate, and official commission following their training. The CHWs were not paid a salary by the study, but received a travel refund after supervision meetings.

While UNEST was initially envisioned to be a community-based intervention, the formative research identified relatively high rates of care-seeking at health facilities with low quality care (18). In response, efforts were made to design the intervention to ensure that all 20 public and private health facilities in and around the study area were strengthened through a 6-day in-service training, provision of a once-off catalytic supply of equipment and medicines, as well as collaboration with the district health team to continuously improve the quality of care provided to mothers and newborns (13, 15). Training modules included goal-oriented ANC, managing maternal complications, infection prevention, managing normal labour and partograph use, neonatal resuscitation, care of the sick newborn, and extra care for the small baby using kangaroo mother care. Space for newborn care, including designated kangaroo mother care beds, was set up in the referral sites. Further details of the health system strengthening are provided elsewhere (13). In the comparison villages, women and their newborns had access to the standard health services, overseen by the district health team, in addition to the improved health facilities.

The trial protocol was approved by Makerere University School of Public Health and the Uganda National Council of Science and Technology. In addition, approval was sought from the district authorities and local leaders in the communities where the study was conducted. The study had a data safety monitoring board comprising local and international maternal and newborn experts which met annually. The trial also had a local advisory board which consisted of academics, national policy and programme managers, and development partners. These met quarterly under the auspices of the Uganda National Newborn Steering Committee. The study was registered as randomised controlled trial ISRCTN50321130.

### Statistical analysis

The number of clusters was fixed *a priori* as the existing villages within the HDSS. Study investigators reviewed data collection tools for accuracy and completeness. Data were double entered in databases with consistency and quality checked. We used an intention to treat approach, where we compared summary variables in the intervention and control arms with adjustment for clustering. Statistical analyses were conducted with Stata version 12. We calculated means and proportions of the background characteristics and compared them with *t* tests or *χ* ^2^ tests as appropriate to assess differences at baseline. Using the *svy* command in Stata (version 12), primary sampling units and strata for the data set were defined to account for the cluster-randomised design. At baseline and endline an asset index score was constructed using principal component analysis to rank households according to asset ownership and then divided into quintiles. Details of how the index was built are described elsewhere (19). The effect of the health system strengthening was assessed using a ‘before–after’ comparison.

## Results

### Care during pregnancy and childbirth

Women's age, parity, and household wealth were similar at baseline across the intervention and control arms (Table 1). Women accessing health facilities for ANC and for delivery services increased in both the intervention and control clusters (Table 2). Whereas almost all women attended ANC at least once in both the intervention (98.9%) and control arm (99.2%) (*p*=0.440), slightly more women did so four or more times in the intervention arm (47.9%) compared to the control arm (43.6%) (*p*=0.165). The median number of ANC visits in both arms was 3. The proportion of women who had at least one home visit during pregnancy was 68.2% in the intervention arm compared to 7.3% in the control arm (*p*<0.001). The median number of pregnancy home visits per woman in the intervention arm was 2.4. The median duration of pregnancy at the time of the first CHW home visit was 4.9 months [standard deviation (SD) 1.9], for the second visit 6.8 months (SD 1.4) and for the third visit 8.0 months (SD 1.3). Women in the intervention arm were more likely to report taking actions to prepare for birth compared to those in the control arm (82.9% vs. 72.7%; *p*=0.003), and this practice more than doubled amongst women in both groups over the course of the study.

**Table 1:** Selected baseline characteristics of women by study arm

	Intervention (*n*=194)		Control (*n*=201)	
	
	*n*	%	*n*	%
Age (years)
<19	16	8.3	20	10.0
19–25	62	32.0	69	34.3
26–30	61	31.4	49	24.4
>30	55	28.4	63	31.3
Parity
1–2	71	36.5	77	38.3
3–4	45	23.2	48	23.9
5 or more	78	40.2	76	37.8
Wealth quintile
Lowest	21	10.8	28	13.9
Second	27	13.9	38	18.9
Middle	52	26.8	36	17.9
Fourth	38	19.6	46	22.9
Highest	26	13.4	28	13.9
Missing	30	15.5	26	12.4

Note: Data are means; percentages are based on cluster averages.

**Table 2:** Care during pregnancy and childbirth

	Intervention		Control		
	
	Baseline (*n*=194) %	Endline (*n*=894) %	Baseline (*n*=201) %	Endline (*n*=893) %	*p*^a^
One or more ANC visits	93.8	98.9	94	99.2	0.440
Four or more ANC visits	29.9	47.0	28.9	43.6	0.165
Prepared for birth^b^	32	82.9	30.9	72.7	0.003
Knowledge of two or more pregnancy-related danger signs	41.8	32.7	41.8	38	0.126
One or more home visit during pregnancy	–	68.2	–	7.3	<0.001
Delivered in health facility	70.6	78.0	68.7	77.7	0.939
Skilled attendant at delivery	58.2	79.6	59.7	78.9	0.826

Note: Percentages based on cluster averages.

a *P*-values calculated with *t*-test to compare differences between intervention and control clusters at endline.

b Birth preparedness refers to acquiring gloves, plastic to deliver on, instruments for cutting and tying cord, cotton wool, and saving money for transport and fees.

The proportion of women who reported having a skilled attendant at delivery did not differ between the intervention and control arm at endline (79.6% vs. 78.9%; *p*=0.826). However, this proportion increased by 21.3 and 19.2% compared to baseline in the intervention and control arm, respectively, and came entirely from increases in use of facilities in the public sector. There was a 13% before–after decrease in women from intervention clusters who reported giving birth in private health facilities (from 29.9 to 16.7%), compared to a 2.5% reduction in the control arms (from 20.4 to 17.9%). Similarly, the use of traditional birth attendants (TBAs) dropped by 5.7% in the intervention arm but remained stagnant in the control arm. Clean delivery practices were high in both arms and did not differ significantly between the intervention and control group.

### Postnatal care contact and practices

Overall 62.8% of the women in the intervention arm were visited by a CHW in the first week after birth compared to 5.8% in the control arm (*p*<0.001). More women who delivered at home and with a TBA were visited by a CHW after birth (73.6%) compared to those who delivered in a hospital or health facility (59.7%) (*p*<0.001) (Table 3).

**Table 3:** Home visits in first week after birth by place of delivery in the intervention arm (*n*=894)

	*n* (%)	*p*^a^
Amongst all births, at least one home visit in the first week after birth	894	
Yes	561 (62.8)	
No	332 (37.1)	
Don't know/missing	1 (0.1)	
Amongst those with at least one home visit in the first week after birth	561	
Babies born at a health facility	416 (59.7)	
Babies born with a TBA/home	145 (73.6)	0.002
Amongst those whose first home visit took place on day 1	228	
Babies born at a health facility	161 (23.4)	
Babies born at home or with a TBA	67 (34.2)	0.004
Women counselled on well baby practices by a CHW after birth, of those who received a home visit	373 (66.5)	
Women counselled on well baby practices by a CHW after birth		
Babies born at a health facility	305 (54.8)	
Babies born at home or with a TBA	64 (32.7)	0.002

Note: Percentages based on cluster averages in the intervention clusters only.

a Calculated with *t*-test (intervention arm vs. control arm at endline).

Of the 561 women in the intervention arm who had a visit by a CHW in the first week after birth, 228 (40.6%) had their first visit on day 1, and only 11.8% or 66 women had their first visit on day 7. Women were more likely to receive a home visit in the first week if they delivered at home (73.6%) compared to those who delivered in a health facility (59.7%) (*p*<0.002). By day 7, the proportion of women visited by a CHW did not differ by place of delivery. Amongst women who gave birth in a health facility and received a CHW home visit in the first week, only 8.2% were visited first on day 1 and 30% were seen on day 2, suggesting that birth in a health facility is associated with a delayed CHW home visit, even though 70% of women were discharged from the facility within 24 hours of delivery.

The proportion of babies who were breastfed within the first hour after birth was significantly higher in the intervention arm compared to the control arm (72.6% vs. 66.0%; *p*=0.0116) (Table 4). This practice increased by 20% from baseline in both arms. The proportion of babies who were exclusively breastfed in the neonatal period was also significantly higher in the intervention arm (81.8%) compared to the control arm (75.9%) (*p*=0.042). Exclusive breastfeeding increased from baseline by 14% and 20% in the intervention and control arm, respectively. The proportion of babies placed skin-to-skin with their mothers immediately after birth was higher in the intervention arm (80.7% vs. 72.2%; *p*=0.071) and amongst those born in health facilities than those born at home or elsewhere.

**Table 4:** Early postnatal practices related to breastfeeding, thermal care, and hygiene

	Intervention				Control				
	
	Baseline (*N*=194)		Endline (*N*=894)		Baseline (*N*=201)		Endline (*N*=893)		
			
	*n*	%	*n*	%	*N*	%	*n*	%	*p*^a^
Optimal feeding
Baby put to the breast within 1 hour of birth	101	52.1	647	72.6	94	46.8	586	66.0	0.016
Baby given colostrum	b	–	835	93.4	b	–	814	91.2	0.086
Baby exclusively breastfed in first month of life	130	67.0	731	81.8	114	56.7	678	75.9	0.042
Thermal protection
Baby placed skin-to-skin with mother within 1 hour of birth	b		721	80.7	b		663	74.2	0.071
Baby dried immediately after birth	b		882	98.7	b		873	97.8	0.754
Baby wrapped immediately after birth	160	82.5	890	99.6	171	85.1	891	99.8	0.562
First bath delayed ≥6 hours after birth	63	32.5	824	92.2	40	19.8	765	85.7	<0.001
First bath delayed ≥24 hours after birth	4	2.06	443	49.6	4	1.99	317	35.5	<0.001
Hygienic care
Cord cut with clean instrument^c^	100	51.6	788	88.1	118	58.7	754	84.4	0.074
Nothing applied to umbilical cord after cutting	81	41.6	571	63.9	114	56.7	474	53.1	0.002

Note: Percentages based on cluster averages.

a Calculated with *t*-test (intervention arm vs. control arm at endline).

b Indicator not collected in baseline survey.

c New blade or boiled blade.

Women in intervention villages reported following better thermal care practices than their counterparts in control areas (Table 4). Almost half (49.6%) of the mothers in the intervention group delayed the baby's first bath for at least 24 hours, compared to 35.5% in the control arm (*p*<0.001). This proportion increased by 47% and 33% between baseline and endline in the intervention and control arms, respectively. The proportion of babies whose bath was delayed for more than 6 hours was also significantly higher in the intervention arm at 92.2% versus 85.7% in the control arm (*p*<0.001). The delayed bathing practice increased by more than 50% in both arms, with no difference in delayed bathing according to place of delivery. Most babies were dried immediately after birth, and this practice was equally high (98.7 and 97.8%) in the intervention and control arms and did not vary between babies born at health facilities or at home. Similarly, the practice of wrapping the baby immediately after delivery was high at baseline but still increased by more than 15% in both arms.

Hygienic cord care practices were better amongst families in the intervention clusters. The proportion of babies whose cord was cut with a clean instrument (new razor blade or boiled tool) was higher in the intervention arm compared to the control arm (88.1% vs. 84.4%; *p*=0.074), but not significantly so (Table 4). The practice increased over the course of the intervention by more than 35% in both arms. The proportion of babies who had nothing put on the umbilical cord stump was 63.9% in the intervention arm, significantly higher than in the control arm at 53.1% (*p*=0.002). This practice increased by 22% from baseline in the intervention arm, whereas it decreased by 3% in the control arm.

Almost all babies reported to have experienced at least one danger sign were taken outside the home for care (95.0% in the intervention arm and 94.1% in the control arm). More than half of all mothers with sick newborns went to a private provider, with drug shops being the first point of care for 24.9 and 28.0% in the intervention and control arms, respectively (*p*=0.412). A total of 162 babies (9.1%) were born with low birthweight (LBW) based on either documentation of baby's weight at birth or the mother's perception that her baby was very small or smaller than average. A significantly higher proportion of LBW babies in the intervention arm were given kangaroo mother care compared to those in the control arm (22.4% vs. 9.3%; *p*=0.089). The proportion of mothers who recognised LBW as a danger sign and sought extra care because of the small size of their baby was higher in the intervention than in the control arm, but the difference was not significant (23.7% vs. 14.0%; *p*=0.234).

### Home visits and CHW workload

Overall CHWs made a total of 4,772 home visits. Each CHW saw an average of 26 mothers per year and made 78 home visits, or 1.5 home visits per week. Each mother and baby received on average 3 of the targeted 5 home visits. The number of CHW visits to mothers with small babies compared to normal weight babies did not vary significantly, implying that LBW babies did not get extra visits – contrary to what the intervention promoted. The average length of a home visit was 82 min, with no significant difference in the time spent by CHWs during either pregnancy or postnatal home visits. Retention of CHWs was 100% during the study implementation period.

## Discussion

Over 2 years of implementation, UNEST achieved significant improvements in birth preparedness and essential newborn care practices, including breastfeeding, hygienic cord care, and thermal protection – practices associated with reduced neonatal mortality. These and other interventions, including ANC and skilled attendance at delivery, recorded increases between baseline and endline. The general improvement in maternal and newborn care practices across both the intervention and control arms may be explained at least in part by the health facility strengthening which impacted both trial arms, but also by the secular trend towards improved maternal and newborn care. CHWs selected by their communities with district-led training and supervision were able to identify and visit almost all the pregnant women, especially those from the poorest families and those who delivered at home or with TBAs. These results have important policy implications in Uganda and in similar settings where CHW programmes for maternal and newborn care are being designed or scaled up.

These findings demonstrate the power of CHWs to effect change in behaviours around maternal and newborn care, and are similar to those reported elsewhere (10, 26). Of the 10 mother-led interventions (birth preparedness, 3 practices related to optimal feeding, 4 thermal care practices, and 2 hygienic cord care practices), 8 reached over 80% coverage in the intervention area. For the intervention and control clusters combined, coverage of the seven practices measured at both baseline and endline was an average of 29% higher post-intervention, demonstrating increased awareness of and demand for newborn care. Contrary to expectations, we found improvements even in practices that formative research indicated may be difficult to change, such as delaying bathing. We are confident that most of the changes observed are mainly due to the CHW intervention, although we are aware some are due to the health facility strengthening, and delivery care is mainly due to a secular trend towards improved institutional births. However, while dry cord care increased and was significantly better in intervention clusters, this coverage remained lowest of all essential newborn care practices, suggesting challenges in improving it. Recent WHO and national recommendations on application of topical antiseptics such as chlorhexidine to the umbilical cord stump (27, 28) may replace the application of common harmful substances, and could be integrated into the CHW messaging during pregnancy and postnatal visits.

Complications of preterm birth are now the second leading cause of all child deaths globally, and the third leading cause in Uganda, after malaria and pneumonia (29). CHW home visits were associated with babies born with LBW receiving kangaroo mother care, demonstrating that awareness during pregnancy is key. In the hospital where kangaroo mother care was introduced, 85% of the 547 babies admitted to the unit were discharged alive (13). Still, this low-tech, caregiver-led intervention reached fewer than 1 in 5 preterm babies. CHWs did not manage to make extra visits to LBW babies, contrary to their training. In order to maximise scalability and limit procurement, the CHWs were not equipped with weighing scales and lacked a reliable mechanism to identify these small babies, particularly those born at home and not weighed at birth. A study nested within UNEST validated a foot length card for use by CHWs (30), which has since been taken up through the national VHT strategy. Future CHW studies must have special attention to care for small babies as a critical part of newborn care.

Care-seeking for routine and extra care services increased in both the control and intervention arms, with an increase in demand for public sector pregnancy and delivery services. ANC and institutional deliveries increased overall, but the proportion of women giving birth at private facilities decreased, with most of the decline amongst women in the intervention arm. Care-seeking for sick newborns was much higher than seen in other settings, but echoes qualitative research indicating that compliance with referral by VHTs is high (31, 32). In contrast to the more common public sector delivery services, private care, mainly through small-scale drug shops, was the first point of service for sick newborns. An assessment of essential newborn care in private facilities in the UNEST areas demonstrated that private health facilities did not perform significantly better than public health facilities, despite the additional cost of these services (33). After facility strengthening, including training and support to management, equipment levels remained high – but maintaining supply of even the most basic medications was a challenge, with less than 40% of health facilities reporting no stock-outs (13). While government engagement is necessary to maintain quality public sector services, strategies are also needed to engage and ensure accountability within the private sector.

Achievements in improved newborn care practices were realised with modest CHW effectiveness in carrying out the intended visit schedule. Only 54.0% of the women in the intervention arm received two or more visits during pregnancy and only 62.8% received a home visit from a CHW in the first week after birth. Even though women were discharged from health facilities within 24 hours, CHWs saw these women later on average than women who delivered at home, perhaps due to lack of notification of delivery and their return home. Mothers in the poorest wealth quintiles were more likely to benefit from these early visits, as were those who delivered at home or with TBAs. While this indicates a desirable pro-poor emphasis, the trend towards increasing institutional deliveries nationally and throughout much of the continent necessitates a platform that works for all women, with stronger communication links to the facilities where women give birth and bring their newborns for care. Here, m-health interventions could be useful as communication tools that allow for support to families at a distance.

While a trial in this well-defined geographical area may represent an efficacy trial, many features of the intervention strategy appear to be scalable with no loss of effectiveness. UNEST utilised district structures to select, train, and supervise CHWs, rather than study staff. Both CHWs and their supervisors did not receive designated salaries, but travel refunds and stipends at a more scalable cost. The cost per mother visited (all visits) stood at US $25 and per home visit at US $8.30. The cost of CHWs is affordable and reduces with scale-up, because the initial set-up costs are not repeated. However, CHW retention was high and more recruitment and retraining would be required over the long term.

The quality and frequency of supervision that can be given by the health workers may vary by setting, and should be taken into consideration. The CHWs hired for UNEST were recruited anew because revitalisation of the national VHT strategy was just beginning when UNEST started. While UNEST CHWs did not have additional responsibilities, their home visit load was generally low and conducive to integration into the broader maternal, newborn, and child health role of the VHTs. Incentivisation and CHW motivation needs to be carefully considered, as reflected in another article in this series (34). To this end the UNEST training package, home visit schedule and behaviour change counselling materials were incorporated into the national VHT and Integrated Community Case Management packages while the study was taking place (35).

One limitation of this study is the differing recall periods of the baseline (4 months) and endline (12 months) surveys for services that occurred before and around the time of birth, although large-scale household surveys include longer recall periods of up to 5 years. An additional limitation is that these surveys only captured women who had live births in both the baseline and endline surveys. The pregnancy home visits, birth-preparedness counselling, and facility quality of care improvements are likely to have had an impact on stillbirths as well as neonatal deaths.

The study was not powered to measure an effect on mortality, although improvements in coverage of care are similar to those seen with a modest mortality impact (10). While the HDSS allowed for documentation of the number and causes of newborn deaths, it was apparent that pregnancy outcomes were being missed in the two census rounds per year. Further analysis and validation of mortality data from household surveys is on-going.

While the improvements in care indicators in both arms could be an effect of the health system strengthening, it could also be an effect of spill-over of the knowledge from one intervention village to the next control village, since there were no buffer villages between intervention and control areas, or a secular trend in Uganda which needs to be corroborated by further research. The scope for potential increase in some of the preventive behaviours was low, because the coverage of these behaviours was already high.

## Conclusion

National attention to newborn survival and health has increased, with UNEST strategically placed to influence key policies and strategies linked to the national VHT strategy and improvements in quality of care at health facilities. Home visits from CHWs are associated with improved essential newborn care practices, regardless of place of delivery. However, there is a key concern around a mismatch between utilisation and quality that results in avoidable deaths.

Additional efforts to prevent the three main causes of neonatal deaths, particularly complications of preterm birth, are needed, linking community efforts to facility quality of care improvement in both the public and private sector. National attention and policies are necessary but not sufficient steps to save newborn lives as well as to prevent maternal deaths and stillbirths. Closing the policy–practice gap at district level is needed to improve maternal and newborn survival and health.
